# Understanding the Added Value of G-Protein-Coupled Receptor Heteromers

**DOI:** 10.1155/2014/362937

**Published:** 2014-04-22

**Authors:** Nuria Franco, Rafael Franco

**Affiliations:** Department Biochemistry and Molecular Biology, Faculty of Biology, University of Barcelona, Prevosti Building, Diagonal 645, 08028 Barcelona, Spain

## Abstract

G-protein-coupled receptors (GPCRs) constitute the most populated family of proteins within the human genome. Since the early sixties work on GPCRs and on GPCR-mediated signaling has led to a number of awards, the most recent being the Nobel Prize in Chemistry for 2012. The future of GPCRs research is surely based on their capacity for heteromerization. Receptor heteromers offer a series of challenges that will help in providing success in academic/basic research and translation into more effective and safer drugs.

## 1. Introduction

Soon in his career, Dr. R. Franco started working with the so-called G-protein-coupled receptors (GPCRs), also known as heptaspanning or serpentine receptors (see below). The “G proteins” coupled to these receptors are called “heterotrimeric” as they are composed of three subunits: *α*, *β*, and *γ*. A very good way to present these receptors is found in the press release for the Nobel Prize in Chemistry for 2012. The awarded scientists were Robert J. Lefkowitz from the Howard Hughes Medical Institute and Duke University Medical Center, Durham, NC, USA, and Brian K. Kobilka from Stanford University School of Medicine, Stanford, CA, USA. The complete press release (from http://www.nobelprize.org/) was as follows.

*Smart Receptors on Cell Surfaces. Your body is a fine-tuned system of interactions between billions of cells. Each cell has tiny receptors that enable it to sense its environment, so it can adapt to new situations. Robert Lefkowitz and Brian Kobilka are awarded the 2012 Nobel Prize in Chemistry for groundbreaking discoveries that reveal the inner workings of an important family of such receptors: G-protein-coupled receptors.*

*For a long time, it remained a mystery how cells could sense their environment. Scientists knew that hormones such as adrenalin had powerful effects: increasing blood pressure and making the heart beat faster. They suspected that cell surfaces contained some kind of recipient for hormones. But what these receptors actually consisted of and how they worked remained obscured for most of the 20th century.*

*Lefkowitz started to use radioactivity in 1968 in order to trace cells' receptors. He attached an iodine isotope to various hormones, and, thanks to the radiation, he managed to unveil several receptors, among those was a receptor for adrenalin: β* *-adrenergic receptor. His team of researchers extracted the receptor from its hiding place in the cell wall and gained an initial understanding of how it works.*

*The team achieved its next big step during the 1980s. The newly recruited Kobilka accepted the challenge to isolate the gene that codes for the β* *-adrenergic receptor from the gigantic human genome. His creative approach allowed him to attain his goal. When the researchers analyzed the gene, they discovered that the receptor was similar to one in the eye that captures light. They realized that there is a whole family of receptors that look alike and function in the same manner.*

*Today this family is referred to as G-protein-coupled receptors. About a thousand genes code for such receptors, for example, for light, flavour, odour, adrenalin, histamine, dopamine, and serotonin. About half of all medications achieve their effect through G-protein-coupled receptors.*

*The studies by Lefkowitz and Kobilka are crucial for understanding how G-protein-coupled receptors function. Furthermore, in 2011, Kobilka achieved another breakthrough; he and his research team captured an image of the β* *-adrenergic receptor at the exact moment that it is activated by a hormone and sends a signal into the cell. This image is a molecular masterpiece—the result of decades of research.*

This paper is written according to what an* Outlook* in Scientifica aims to be, that is, a brief review (with limited amount of references) on a number of closely related projects in a specific laboratory (in this particular case a laboratory that has been working for years in GPCRs) and a forward-looking analysis of the issues that authors believe will drive future research on the specific field and topic. The paper is written with a historical perspective that may help in understanding the advances in the GPCR heteromer field.

## 2. Adenosine, Adenosine Deaminase, and Adenosine Receptors

The smart receptors chosen back in 1985 at the Department of Biochemistry of the University of Barcelona were adenosine receptors and the reason is simple. First of all, R. Franco did his Ph.D. studying the purine metabolic enzymes in brain and one of the most important findings was the ectoenzyme nature of adenosine deaminase [1] that was later proven from blood cells [2] to neurons [3]. The enzyme removes extracellular adenosine, which is there (extracellularly) to activate cell surface adenosine receptors. However, a stay in the laboratory of Professor G. Burnstock, at the University College of London at that time, was needed to notice that GPCRs were worth being devoted a scientific career. It is true that Professor Burnstock's interests were more on knowing molecular aspects of ATP neurotransmission, but ATP receptors were for a young scientist a big challenge. It was safer to opt for investigating the more modest adenosine receptors for which good tool compounds (agonists and antagonists) started to be available. Tool compounds for ATP receptors have been much more difficult to obtain and, also, ATP receptors can be ionotropic (P2X) and metabotropic (P2Y) and there are a total of about 20 different subtypes plus a significant number of splice variants [4]. Adenosine receptors are only four and few splice variants have been described and/or characterized. In the eighties adenosine receptors were divided into two: the one whose activation leads to increases in cAMP (A_1_) and the one that leads to decreases in cAMP (A_2_) [5]. Different pharmacological profiles among A_2_ receptors led to postulate the existence of two different A_2_ receptors: A_2A_ and A_2B_ [6, 7]. When many GPCRs were cloned, four different adenosine receptors were identified and named as follows: A_1_, A_2A_, A_2B_, and A_3_.

## 3. G-Protein-Coupled Receptors Also Interact with Other Proteins

Definition of GPCRs as coupled to G protein is somewhat misleading as the receptors may bind other proteins and G-protein-independent events also happen upon GPCR activation. In the Molecular Neurobiology Laboratory of the University of Barcelona, a number of proteins were identified as able to interact with the A_1_, A_2A_, and/or A_2B_ receptors. To the surprise of all scientists in the whole laboratory, one of them was adenosine deaminase, which is routinely added to all assays to get rid of endogenous adenosine. The ectoenzyme nature of adenosine deaminase (ADA1) was confirmed by Kameoka et al. [8], who reported that an activation lymphocyte marker, CD26, was able to bind ADA1 on the cell surface [9]. On the one hand, extracellular ADA1, including that circulating in the blood, may bind to a variety of membrane proteins (adenosine receptors [10–12] and CD26). On the other hand, the binding of the enzyme that degrades a neuromodulator or neurohormone, adenosine, to an adenosine receptor posed a conceptual problem. Different experimental approaches led to showing that the interaction of the two proteins confers high affinity for adenosine, that is, that low concentration of adenosine leads to efficient A_1_ receptor activation. At higher concentrations ADA is no longer interacting with the receptor that in turn becomes refractory to the compound. Further work ended up with a nice model by which ADA1 may act as a bridge between two cells, one displaying CD26 and the other displaying A_1_ receptors.

ADA1 is a protein whose deficit leads to severe combined immunodeficiency. It may bind not only to CD26 and A_1_ adenosine receptors but also to A_2A_ or A_2B_ adenosine receptors. Pacheco et al. [13] reported that ADA1 “*enhanced T cell proliferation in autologous co-cultures with antigen-pulsed immature or mature dendritic cells. They also showed a 3-fold reduction in the EC*_*50*_ *for the antigen by the action of ADA1.*” Furthermore, costimulation was not due to the enzymatic activity but to the interaction of ADA-CD26 complexes in T cells. In fact ADA1 acted as a bridge by a dual interaction, with adenosine receptors expressed on dendritic cells and with CD26 expressed on lymphocytes. This cell-to-cell communication facilitated by ADA1 constitutes an important component in the immunological synapse and leads to a marked increase in the production of diverse and physiologically relevant cytokines [14]. For other adenosine-receptor-interacting proteins that were identified in our laboratory, see the following: Sarrió et al. [15]; Burgueño et al. [16]; Canela et al. [17]; Franco et al. [18, 19].

## 4. Homodimers and Homooligomers

Starting around the mid-nineties, the focus of the Molecular Neurobiology Laboratory has been devoted to understand why many GPCRs, adenosine receptors included, form dimers and even higher-order structures. With exceptions, homodimers seem to be the most predominant form of GPCRs on the cell surface, which is where the receptor meets the hormone/neurotransmitter [20]. The occurrence of homodimers instead of monomers may not be relevant from a physiological point of view. In fact monomers of receptors may signal, at least in artificial membranes able to incorporate GPCRs. Whorton et al. [21] developed high-density lipoprotein phospholipid bilayer particles containing beta2-adrenergic receptors and the stimulatory heterotrimeric G protein, Gs. A monomeric adrenergic receptor molecule may activate Gs and display GTP-sensitive allosteric ligand-binding properties thus suggesting that a monomeric receptor is the minimal functional unit necessary for signaling in a lipid bilayer.

It should be noted that metabotropic glutamate receptors, which belong to another subfamily of GPCRs-class C-, tend to form homodimers in which a ligand binds to one of the protomers, but the dimer signals through the G protein that is coupled to the second protomer [22]. It may occur that a given G protein sits below a dimer but in asymmetrical form, that is, having the *α* subunit of the G protein close to one of the protomers but not to the other. In any case the occurrence of dimers may be irrelevant if one's focus is a given receptor and a given signaling pathway. However dimers open a completely new pharmacological field that helps to analyze data and interpret them. For instance, radioligand binding of agonists to the A_1_ receptor leads to two different affinities. Classically they have been considered high- and low-affinity forms of the receptor, the high-affinity one assumed to be due to the G-protein-receptor complex and the low-affinity one assumed to be due to the receptor (without the G protein). As dimers have two different binding sites, one in each protomer, it might be that each binding site has its own affinity for a given ligand; in other words, the binding of the first molecule to one of the protomers may lead to conformational changes in the dimer that affects the binding to the second (empty) binding site. Drs. Enric I. Canela and Vicent Casadó were able to build up the* two-state dimer model* and develop the tools for fitting binding data to receptor dimers. The model is simpler than that assuming the occurrence of receptor with and without G proteins and made predictions that were demonstrated in* in vitro* assays [23, 24].

## 5. Intramolecular Conformational Changes

The phenomenon of ligand-driven conformational changes in oligomeric proteins, that is very familiar for enzymologists and protein biochemists and is known by them as “cooperativity,” is now incorporated into pharmacology. As hemoglobin needs to be constituted by 4 protomers to provide advantages to mammals, GPCRs may need to form dimers or higher-order oligomers for optimal biological performance. To understand whether homooligomers have advantages over monomers is however not an easy task. The task is a bit easier when considering heteromers.

## 6. Heterodimers and Heterooligomers

Advance on understanding the biology of GPCR heteromers is nowadays a challenge in the field and the main objective in the laboratory. The work ahead is straightforward and needs to respond to an apparent simple question, which is the added value of heteromers in nature. It should be noted that to convince scientists of the occurrence of such heteromers has not been an easy task. Fortunately there is now consensus [25] on the presence of heteromers in almost every cell in every tissue.

## 7. Added Value of Heteromers

**Figure 1:**
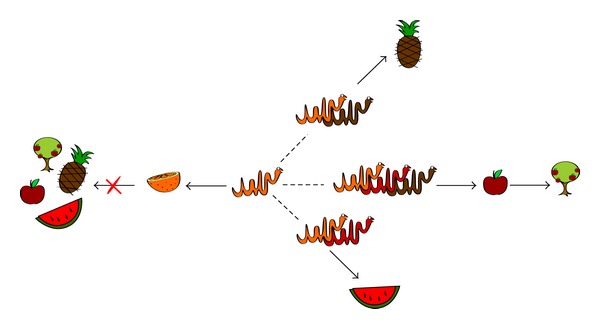
Cartoon showing that cooperation between different receptors may achieve objectives that are unobtainable for a single receptor. In a tribute to one of the names used for these receptors (serpentine receptors) they are depicted as serpents/snakes; each colored serpentine would correspond to a given GPCR. The fruits and the fruit tree are a pictorial metaphor of cell/biological functions that may be reached by activation of GPCR monomers, dimers, or trimers. Copyright is retained by N. Franco.

From work in different laboratories it becomes clear that GPCR heteromers confer selective advantages. Whereas monomeric (even homomeric) receptor operation is monotone, heteromeric receptor operation is multiple and varied.  Figure 1 displays a cartoon showing the advantages of receptor cooperation due to heteromerization. Structurally GPCRs have seven transmembrane domains with extracellular and intracellular loops connecting them, plus the extracellular N terminal domain and a cytoplasmic C terminal tail. Due to this particular structure, these receptors are also known as heptaspanning or serpentine receptors. The abbreviation “sr” for serpentine is being used for the subfamilies (sra, srb, etc.) of the numerous chemosensory GPCR receptors in* Caenorhabditis elegans* [26]. This is the reason for displaying GPCRs in Figure 1 as serpents.

A few examples of heteromer-directed function, coming from the work in the Laboratory of Susan George (University of Toronto) and from that in the University of Barcelona, will be here provided. The D_1_ dopamine receptor subtype is coupled to Gs proteins, that is, to proteins that mediate increases in cAMP. In contrast, the D_2_ subtype is coupled to Gi proteins, which mediate decreases in cAMP. Interestingly D_1_-D_2_ receptor heteromers do not couple with Gs or Gi but with Gq, which trigger calcium mobilization. These results are important as they show that a single neurotransmitter, dopamine, may lead to changes in cAMP in D_1_-receptor-containing neurons or D_2_-receptor-containing neurons or to increases in intracellular calcium in neurons that contain D_1_-D_2_ receptor heteromers. A single neurotransmitter may thus trigger three different responses in three different cells or, eventually, in three different subcellular locations of the same cell. Dopamine D_1_ and D_2_ receptors are the most abundant dopaminergic receptors in the striatum, and although a clear segregation between the pathways expressing these two receptors has been reported in certain subregions, the presence of  D_1_-D_2_ receptor heteromers occurs within a unique subset of neurons; in these cells heteromers form a novel signaling transducing functional entity. Recently, significant progress has been made in elucidating the signaling pathways activated by the D_1_-D_2_ receptor heteromers and their potential physiological relevance [27]. In fact the Gq-mediated signaling cascade may be activated in adult rat brain, although with regional specificity (largely limited to the* nucleus accumbens*). This specific dopaminergic pathway seems to regulate brain-derived neurotrophic factor effects on neuronal growth and maturation, thus having considerable interest in disorders such as drug addiction, schizophrenia, and depression [28, 29]. May dopamine via, for instance, D_2_ receptor, trigger a further response? Indeed it is possible as it has been shown that these receptors may form trimers with A_2A_ receptor and with cannabinoid CB_1_ receptors and that their activation engages another heteromer-specific signaling pathway [30, 31].

A different example is that constituted by the A_1_-A_2A_ receptor heteromers, which arise as a sensor of the concentration of extracellular adenosine. Ciruela et al. [32] demonstrated that heteromerization of adenosine A_1_ and A_2A_ receptors allows adenosine to exert a fine-tuning modulation of glutamatergic neurotransmission. Using a convergent approach using coimmunoprecipitation, bioluminescence, and time-resolved fluorescence resonance energy transfer techniques, A_1_-A_2A_ receptor heteromers were detected in the cell surface of cotransfected cells. Immunogold detection and coimmunoprecipitation experiments indicated that A_1_ and A_2A_ are colocalized in the same striatal glutamatergic nerve terminals. Radioligand-binding experiments in cotransfected cells and rat striatum showed that a main biochemical characteristic of the A_1_-A_2A_ heteromers is the ability of A_2A_ receptor activation to reduce the affinity of the A_1_ receptor for agonists. Overall the heteromer provides a switch mechanism by which low and high concentrations of adenosine inhibit and stimulate, respectively, glutamate release.

Interestingly the A_1_-A_2A_ receptor heteromers are also expressed in astrocyte populations [33]. Astrocytes play a key role in modulating synaptic transmission by controlling extracellular gamma-aminobutyric acid (GABA) levels via GAT-1 and GAT-3 GABA transporters. Using primary cultures of rat astrocytes, Cristóvão-Ferréira et al. [33] showed that a further level of regulation of GABA uptake occurs by activation of adenosine receptors in the A_1_-A_2A_ receptor heteromers. Adenosine regulation of GABA uptake occurs via two different G proteins, Gs and Gi/o, and the nucleoside either enhances (A_2A_) or inhibits (A_1_) GABA uptake. Again an opposite regulation is possible by just one molecule, adenosine, and acts on A_1_-A_2A_ receptor heteromers. This would not be possible if neurons or astrocytes express A_1_ or A_2A_ or a combination of A_1_ and A_2A_ receptors. The characteristics of the A_1_-A_2A_ receptor heteromers are unique and do not merely result from the addition of two independent receptor-mediated signals.

## 8. Heteromers in Natural Tissues and in Human Disease

AbdAlla et al. [34] were the first to link GPCR heteromers and disease; they suggested that angiotensin 1-bradykinin 2 receptor heterodimers contribute to angiotensin II hypersensitivity in preeclampsia. Thereafter not many other disorders have been associated with altered G-protein-coupled receptor heteromerization. Understanding the factors regulating heteromer expression and whether pathological conditions affect heteromer expression are important challenges in the GPCR heteromer field. There are still difficulties in knowing whether heteromers are present in natural tissues. Until recently, heteromers in natural sources could be detected by looking for a fingerprint. For instance, synthetic radioligand binding to a receptor in a given heteromer is usually modified if the partner receptor is occupied by a selective synthetic ligand. A novel and powerful technique is now available and consists of detecting heteromers in slices from natural tissues using specific antibodies and an appropriate amplification approach. The technique is known as* in situ* proximity ligation assay. Very recently we have shown that heteromers are expressed in nonhuman primate (*Macaca fascicularis*) striatum and in the 1-metil-4-fenil-1,2,3,6-tetrahidropiridina- (MPTP-) primate model of Parkinson's disease [35]. By using the* in situ* proximity ligation assay and by identification of a radioligand-based fingerprint, A_2A_-CB_1_-D_2_ receptor heteromers were found in the* caudate-putamen* of naïve and MPTP-treated animals. Interestingly, L-3,4-dihydroxyphenylalanine (L-DOPA) treatment blunted the biochemical fingerprint and led to weak heteromer expression; these findings constitute the first evidence of altered receptor heteromer expression due to therapy and suggest that drugs specifically targeting A_2A_-CB_1_-D_2_ receptor heteromers may be successful in either normalizing basal ganglia output or preventing L-DOPA-induced side effects.

## 9. Heteromers as Potential Targets in Human Therapy

GPCRs have been successful as targets for human therapy. Adenosine receptors in general and A_1_ and A_2A_ receptors in particular are potential targets for a variety of diseases, from arrhythmias [36, 37] to Alzheimer's disease [38]. As these receptors may form heteromers, it is time to think about heteromers themselves as potential targets. For sure (see Section 8) heteromers are present in parkinsonian conditions; it is therefore evident that current drugs are targeting these heteromers; that is, L-DOPA treatment in parkinsonian patients is targeting dopamine receptors both in “monomeric” and “heteromeric” form. The possibility to differentially target some heteromers and not others is very attractive (see below). Not very often the binding of the endogenous hormone/neurotransmitter affects the binding of the endogenous hormone/neurotransmitter in the partner receptor. In contrast, synthetic ligands may preferentially target a given receptor in the conformation of a particular heteromer. At present heteromer-selective compounds have been mainly found by serendipity and/or by testing already existing molecules in different heteromeric contexts. The future will tell whether screening for heteromer-selective compounds will be common practice in pharmaceutical companies and/or in medicinal chemistry laboratories. A couple of approaches to facilitate screening for heteromers based on time-resolved fluorescence resonance energy transfer are currently being tested in the Laboratory of the University of Barcelona.

## 10. Targeting Pre- or Postsynaptic Heteromers

Striatal adenosine A_2A_ receptors are highly expressed in medium spiny neurons of the one of the two main basal ganglia circuits (the indirect efferent pathway), where they heteromerize with dopamine D_2_ receptors; A_2A_ receptors are also localized presynaptically in corticostriatal glutamatergic terminals contacting medium spiny neurons of the second efferent pathway (the direct pathway), where they heteromerize with adenosine A_1_ receptors [39, 40]. It has been hypothesized that presynaptic A_2A_ receptor antagonists could be beneficial in dyskinetic disorders (Huntington's disease) while postsynaptic A_2A_ receptor antagonists may be useful in Parkinson's disease. On checking whether selective A_2A_ receptor antagonists may be subdivided according to a preferential pre- versus postsynaptic mechanism of action, Orru et al. [41] reported that two antagonists, SCH-442416 and KW-6002, had a significant preferential pre- and postsynaptic profile, respectively, while other tested compounds (MSX-2, SCH-420814, ZM-241385, and SCH-58261) showed no clear preference. By radioligand-binding experiments performed in cells expressing A_2A_-D_2_ or A_1_-A_2A_ receptors, it is possible to show that heteromerization plays a key role in the presynaptic profile of SCH-442416, which binds with much less affinity to A_2A_ receptors when coexpressed with D_2_ than when coexpressed with A_1_ receptors. KW-6002 showed better relative affinity for A_2A_ receptors coexpressed with D_2_ receptors than coexpressed with A_1_ receptors, which can at least partially explain the postsynaptic profile of this compound. Also, the* in vitro* pharmacological profile of MSX-2, SCH-420814, ZM-241385, and SCH-58261 was in accordance with their mixed pre- and postsynaptic profile. These results pave the way to develop preferential pre- versus postsynaptic actions or vice versa. In the case of A_2A_ receptor antagonists, this heteromer-selective screening would lead to either antidyskinetic or antiparkinsonian drugs. It should be noted that in 2013 KW-6002 (istradefylline) has been approved in Japan as antiparkinsonian agent (sold as Nouriast by Kyowa Hakko Kirin Co., Ltd.). The compound reduces dyskinesia resulting from long-term treatment with classical drugs such as L-DOPA see http://www.kyowa-kirin.com for further information. In fact drugs targeting heteromers with high affinity would be safer as a given heteromer is only expressed in very precise locations and the expected drug doses should be smaller than current ones.
